# Rosmarinic Acid Restores Complete Transparency of Sonicated Human Cataract *Ex Vivo* and Delays Cataract Formation *In Vivo*

**DOI:** 10.1038/s41598-018-27516-9

**Published:** 2018-06-19

**Authors:** Marina Chemerovski-Glikman, Michael Mimouni, Yarden Dagan, Esraa Haj, Igor Vainer, Raviv Allon, Eytan Z. Blumenthal, Lihi Adler-Abramovich, Daniel Segal, Ehud Gazit, Shiri Zayit-Soudry

**Affiliations:** 1 Department of Molecular Microbiology and Biotechnology, George S. Wise Faculty of Life Sciences, Tel-Aviv University, Tel Aviv, 69978 Israel; 2 Department of Ophthalmology, Rambam Health Care Campus, Technion Israel Institute of Technology, Haifa, Israel; 3 Department of Oral Biology, The Goldschleger School of Dental Medicine, Sackler Faculty of Medicine, Tel Aviv University, Tel Aviv, 69978 Israel; 4 Sagol Interdisciplinary School of Neurosciences, Tel-Aviv University, Tel Aviv, 69978 Israel; 5 Department of Materials Science and Engineering, Iby and Aladar Fleischman Faculty of Engineering, Tel Aviv University, Tel Aviv, 6997801 Israel

## Abstract

Cataract, the leading cause of vision impairment worldwide, arises from abnormal aggregation of crystallin lens proteins. Presently, surgical removal is the only therapeutic approach. Recent findings have triggered renewed interest in development of non-surgical treatment alternatives. However, emerging treatments are yet to achieve full and consistent lens clearance. Here, the first *ex vivo* assay to screen for drug candidates that reduce human lenticular protein aggregation was developed. This assay allowed the identification of two leading compounds as facilitating the restoration of nearly-complete transparency of phacoemulsified cataractous preparation *ex vivo*. Mechanistic studies demonstrated that both compounds reduce cataract microparticle size and modify their amyloid-like features. *In vivo* studies confirmed that the lead compound, rosmarinic acid, delays cataract formation and reduces the severity of lens opacification in model rats. Thus, the *ex vivo* assay may provide an initial platform for broad screening of potential novel therapeutic agents towards pharmacological treatment of cataract.

## Introduction

Cataract, defined by abnormal opacification of the intraocular crystalline lens, is the leading cause of blindness in the developing world, affecting millions worldwide^1^. The number of people blind from bilateral cataracts, currently estimated at 20 million, is projected to increase with the rising life expectancy^1,2^. Currently, surgical removal of the opaque lens is the only available treatment for clinically significant cataract. Modern techniques yield high rates of success in restoring visual function and improving quality of life^3^. Nonetheless, this surgery is unattainable for a large portion of the world’s population, especially in under-developed countries where access to surgical ophthalmic care is currently limited^4^. Moreover, in spite of the general high safety of well-established advanced techniques employed in modern cataract surgery, various intraoperative and postoperative complications may occur in approximately 5% of the patients^5,6^. Thus, devising pharmacological treatment for cataract may help amend the global morbidity associated with this major public health concern.

Whereas the physiological transparency of the lens results from a well-organized supramolecular arrangement of the crystallin proteins, including the α, β, and γ- crystallins^7^, loss of lenticular clarity arises from their pathological aggregation^8^ and formation of amyloid assemblies^9^. Attempts have matured in recent years to develop pharmacological treatments for cataract that facilitate restoration of the crystalline lens transparency by systemic^10–13^ and topical administration^14–17^. For example, lanosterol was reported to reduce aggregation of various crystallin proteins *in vitro* and decrease cataract severity in a canine model^15^. Another sterol, 25-hydroxycholesterol, was reported to inhibit α-crystallin aggregation and reverse this process *in vitro* as well as to partially restore protein solubility in an age-related mouse cataract model and in whole human lenses *ex vivo*^16^. However, doubts have been raised regarding the ability to achieve full transparency of the human lens in eyes with cataract. Specifically, sterol treatment did not result in full clarity of the cataractous material^15,16,18^. Hence, there is still an unmet need for such cataract modulating agents.

We postulated that targeting lenticular protein aggregates would reduce their load of precipitates and would affect their light-scattering properties, thus ameliorating cataract. To address this hypothesis, we have developed a novel *ex vivo* platform in which human lens particles removed from patients during routine cataract surgery were treated with one of several protein aggregation modulators. This simple yet innovative experimental approach has enabled us to directly test the impact of the screened compounds on protein aggregates present in phacoemulsified human crystalline lens material. To date, this is the first reported use of *ex vivo* human cataract material for the systematic screening of potential therapeutic agents.

Our findings confirmed the reported efficacy of 25-hydroxycholesterol in reducing cataractous protein load^16^, validating the utility of our assay. However, as previously reported, complete optical clearance of the treated solution was not observed using this compound. By employing our novel screening method, we further demonstrated that rosmarinic acid and doxycycline are potent cataract modulators showing better optical clearance and reduction of amyloid content *ex vivo* as compared to sterols. *In vitro* methods were further applied to derive mechanistic insights on the disaggregation effects of these compounds. Moreover, treatment with rosmarinic acid deterred cataractogenesis in model rats, providing a proof of concept that modulation of protein aggregation can ameliorate cataract formation *in vivo*. These results provide conceptual and mechanistic insights into the development of novel therapeutic strategies for vision loss caused by cataract, and support the utility of the *ex vivo* platform for initial testing of the efficacy of potential cataract modulating agents.

## Results

### Extraction and characterization of human cataractous material

**Figure 1:**
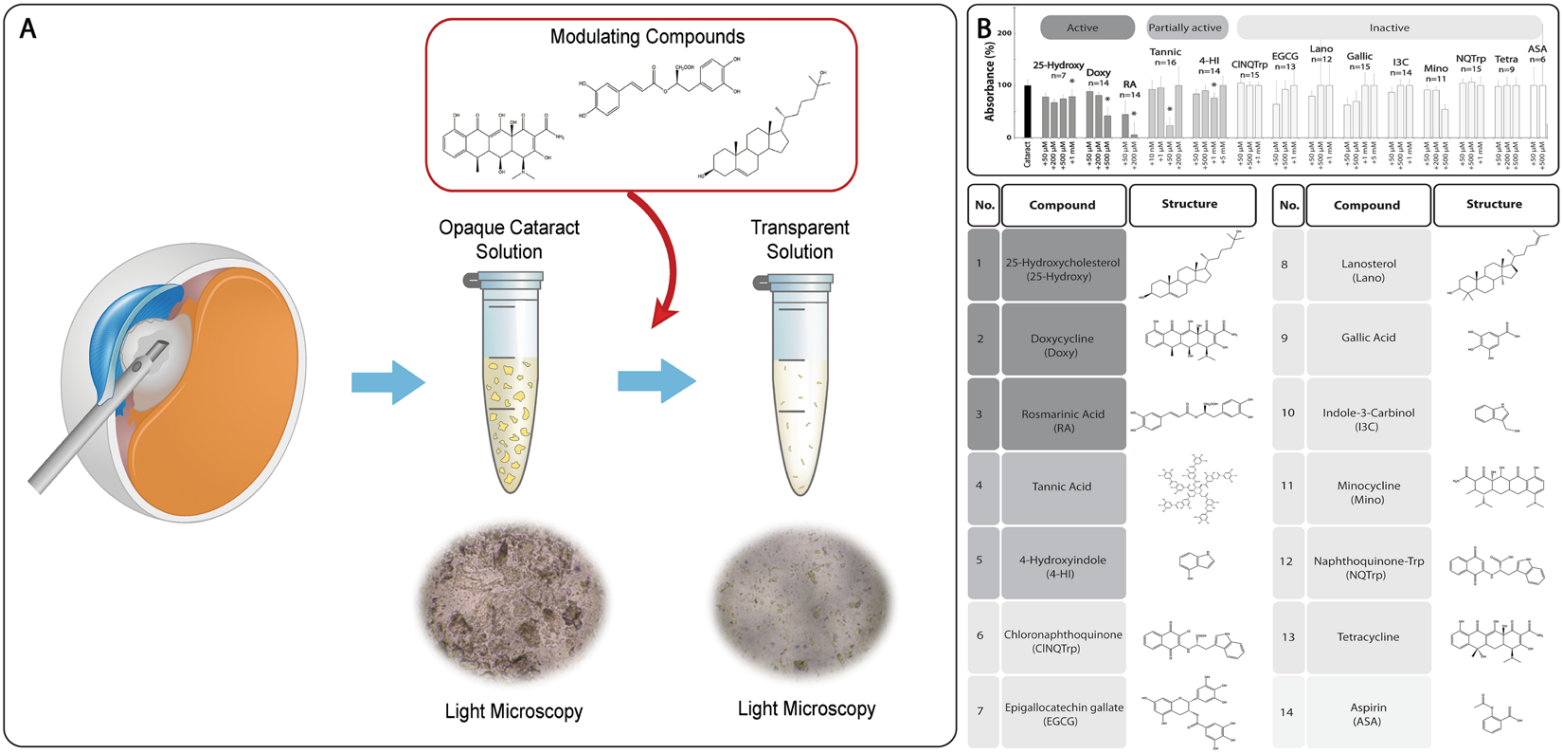
*Ex vivo* assay for screening of human cataract modulating compounds. (**A**) Schematic outline of the screening system. Human cataract lens fragments were removed from patients undergoing routine cataract surgery. Solutions containing the cataract material were then exposed to modulating compounds and the results were examined. (**B**) Non-biased screening of known amyloid modulators for a cataract modulating effect was performed. Colors correspond to the effect on turbidity of cataract solutions: dark gray: active; medium gray: partially active; light gray: inactive. The disaggregation of human cataract samples was monitored by absorbance measurement at λ = 340 nm and 37 °C. Columns denoted by * indicate statistical significance of the results.

To obtain an experimental model which allows to directly determine the impact of aggregation modulators on human cataract (Fig. 1A), nuclear lens fragments removed from patients undergoing routine cataract phacoemulsification surgery were collected from 80 eyes of 80 subjects with age-related cataract (mean age 73.4 ± 9.3 years, 48% male).

**Figure 2:**
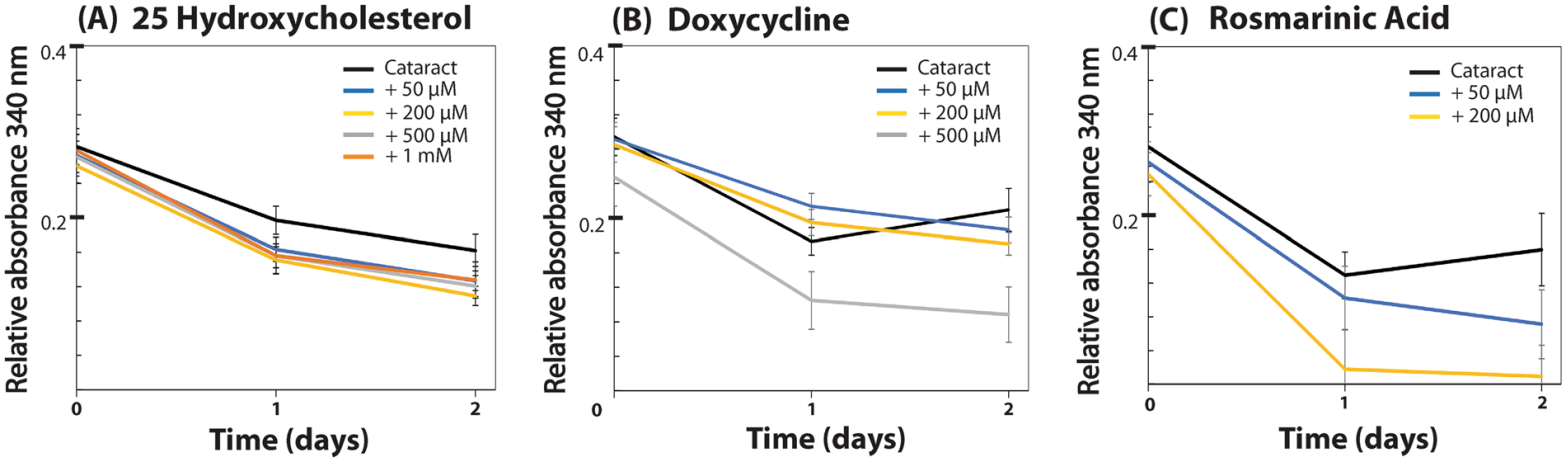
Human cataract disintegrating compounds identified by *ex vivo* turbidity screening. The disaggregation of human cataract samples was monitored by absorbance measurement at λ = 340 nm and 37 °C. Cataract solutions were incubated with increasing concentrations of (**A**) 25-hydroxycholesterol, (**B**) doxycycline, and (**C**) rosmarinic acid in triplicates, for two days, and the turbidity was measured daily. All three compounds reduced cataract turbidity in a dose-dependent and significant manner.

Dispersed nuclear cataract material from each patient was individually incubated with increasing concentrations of each of the tested compounds under shaking conditions at 37 °C over 2 days. The optical density of the solution at 340 nm was measured daily. 25-hydroxycholesterol, which was independently reported during the development of our assay to improve lens transparency *ex vivo* and in an animal model^16^, was used as a positive control. Figures 1B and 2A show that treatment with 1 mM 25-hydroxycholesterol resulted in ~20% reduction of cataract solution turbidity (n = 7, **P = 0.02). Interestingly, lanosterol, which was reported to reverse protein aggregation in a canine cataract model^15^, did not reduce the turbidity of the examined human cataract samples (Fig. 1B). This observation is however in agreement with a more recent study, in which lanosterol had no apparent effect on the opacification of human cataractous lens nuclei^18^. Aspirin, not known to affect protein aggregation^19^, was used as a negative control and indeed did not alter the turbidity of the cataract samples (Fig. 1B).

Next, under the same experimental conditions, a non-biased screen of known amyloid modulators was performed, including minocycline^20^, tetracycline^21^, 4-hydroxyindole, indol-3-carbinole^22^, epigallocatechin gallate (EGCG)^23,24^, gallic acid^25^, tannic acid^26^, 1,4-naphthoquinon-2-yl-L-tryptophan (NQTrp)^27^, and N-(3-chloro-1,4-dihydro-1,4-dioxo-2-naphthalenyl)-L-tryptophan (ClNQTrp)^28^. None of these compounds demonstrated a significant dose-dependent effect on the turbidity of the cataractous solutions (Fig. 1B). Although tannic acid was reproducibly potent at 50 μM, a dose-response relationship was not demonstrated for this compound. This can be explained by the fact that at the higher concentrations tested (200 and 500 μM), tannic acid can intrinsically self-assemble into ordered structures (Fig. S1), thus potentially confounding its effect on the turbidity of the solution.

Notably, our screening method revealed two compounds which reduced the turbidity of the sonicated phacoemulsified cataractous solutions: doxycycline and rosmarinic acid (Fig. 2B,C). Both of these small molecules, also known for their amyloid destabilization effect^21,29–31^, showed a unique and dynamic clearance pattern that was significant and dose-dependent. Treatment with 500 µM doxycycline resulted in ~60% reduction of the turbidity (versus ~25% with vehicle only) (n = 14, **P = 0.03), while treatment with 200 µM of rosmarinic acid led to ~90% reduction (versus ~35% with vehicle only) in the turbidity of cataract samples (n = 14, **P ≤ 0.001). Control wells contained equivalent concentrations of the compounds in sterile balanced saline solution (BSS) and were measured in parallel for subtraction of background turbidity. In between measurements, plates were kept sealed at 37 °C with constant shaking.

To obtain control turbidity measures from clear crystalline lenses, and to examine the effect of the lead compounds (rosmarinic acid, doxycycline and 25-hydroxycholesterol) on lens proteins from eyes untamed with cataract, freshly harvested (n = 5) clear crystalline bovine lenses were treated in a similar fashion to the cataractous human lens samples (Fig. S2). The sonicated lens protein solutions were treated with the lead compounds in concentrations identical to those employed for the *ex-vivo* human cataract experiments. Indeed, sonicated intact crystallins from clear bovine lenses do not necessarily mimic the aggregation state potentially present in phacoemulsified cataract from aged human lenses. Nonetheless, no significant difference in reduction in the turbidity of the sonicated clear bovine lens particles was observed over 2 days after treatment with either compound when compared to the vehicle only (Fig. S2). The slight reduction in the turbidity of the control bovine lens samples may hypothetically reflect processes of disintegration as supramolecular structures may be present also in a normal eye. However, the minor reduction seen in turbidity was to a much lower extent as compared to the effect noted in the phacoemulsified human cataractous preparation.

### Biophysical characterization of treated cataract samples

**Figure 3:**
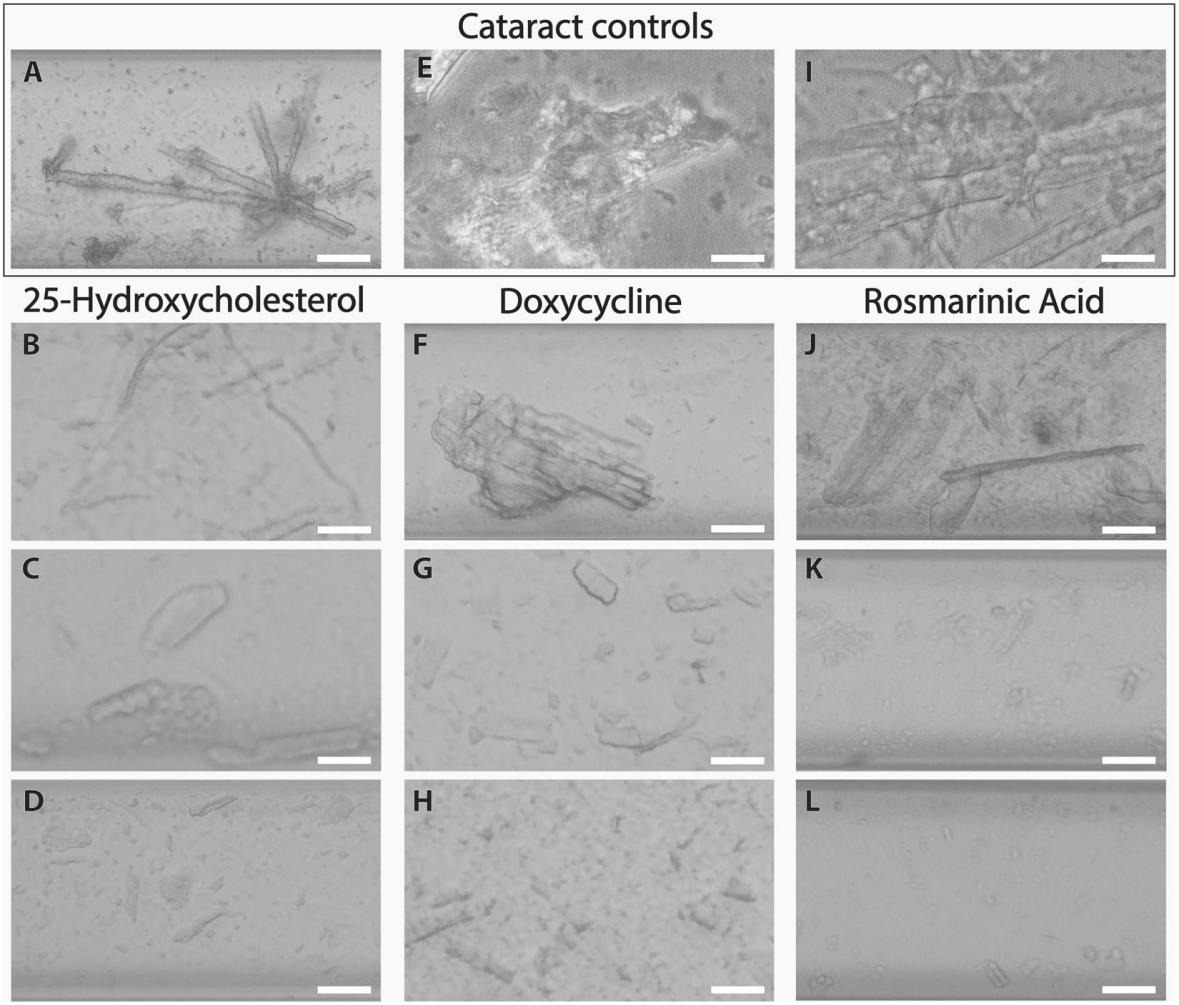
Morphological characterization of cataract disintegration by 25-hydroxycholesterol, doxycycline, and rosmarinic acid. Bright field image sequences of cataract particles in 10% DMSO (**A**–**D**) or pure BSS (**E**–**L**), in the absence (**A**,**E**,**I**) or presence of increasing concentrations (50, 500 μM, and 1 mM) of 25-hydroxycholesterol (**B**–**D**), doxycycline (**F**–**H**), or rosmarinic acid (**J**–**L**) are shown. Samples were transferred to a glass capillary after 2 days of incubation, sealed, and viewed under light microscopy. Scale bars, 100 µm. Representative bright field images from three different experiments conducted with each compound are shown.

We further aimed to gain insight into the morphological transformation induced in the human lenticular protein aggregates by the identified lead compounds. Since all dispersed cataractous solutions appeared macroscopically clouded, we employed light microscopy to assess the *ex vivo* lens material and the effect of the treatment on its morphology. Various cataract solutions were incubated for two days with increasing concentrations of 25-hydroxycholesterol, doxycycline, or rosmarinic acid and subsequently inspected under bright field microscopy. Non-treated human cataract particles appeared as containing microparticles at the micron-range (Fig. 3A,E,I). Although such structures may in theory represent aggregates generated by the phacoemulsification, it is assumed that these were actual cataract fragments. These structures were also observed in the presence of the lowest tested concentration (50 µM) of each of the identified aggregation modulators (Fig. 3B,F,J). In the presence of 500 µM (Fig. 3C,G,K) and 1 mM (Fig. 3D,H,L) of 25-hydroxycholesterol, doxycycline, or rosmarinic acid, there was a clear reduction in the abundance and size of the cataract particles. This effect was especially noticeable upon treatment with doxycycline (Fig. 3G,H) and rosmarinic acid (Fig. 3K,L), both leading to disintegration of the aggregates into particles smaller than those seen in the presence of 25-hydroxycholesterol (Fig. 3B,C,D). Notably, rosmarinic acid demonstrated the strongest effect at the highest tested concentration (Fig. 3L), leading to near complete disintegration of the preparations. None of the control solutions of 25-hydroxycholesterol, doxycycline, or rosmarinic acid examined under the same conditions demonstrated any structural or ordered characteristics.

**Figure 4:**
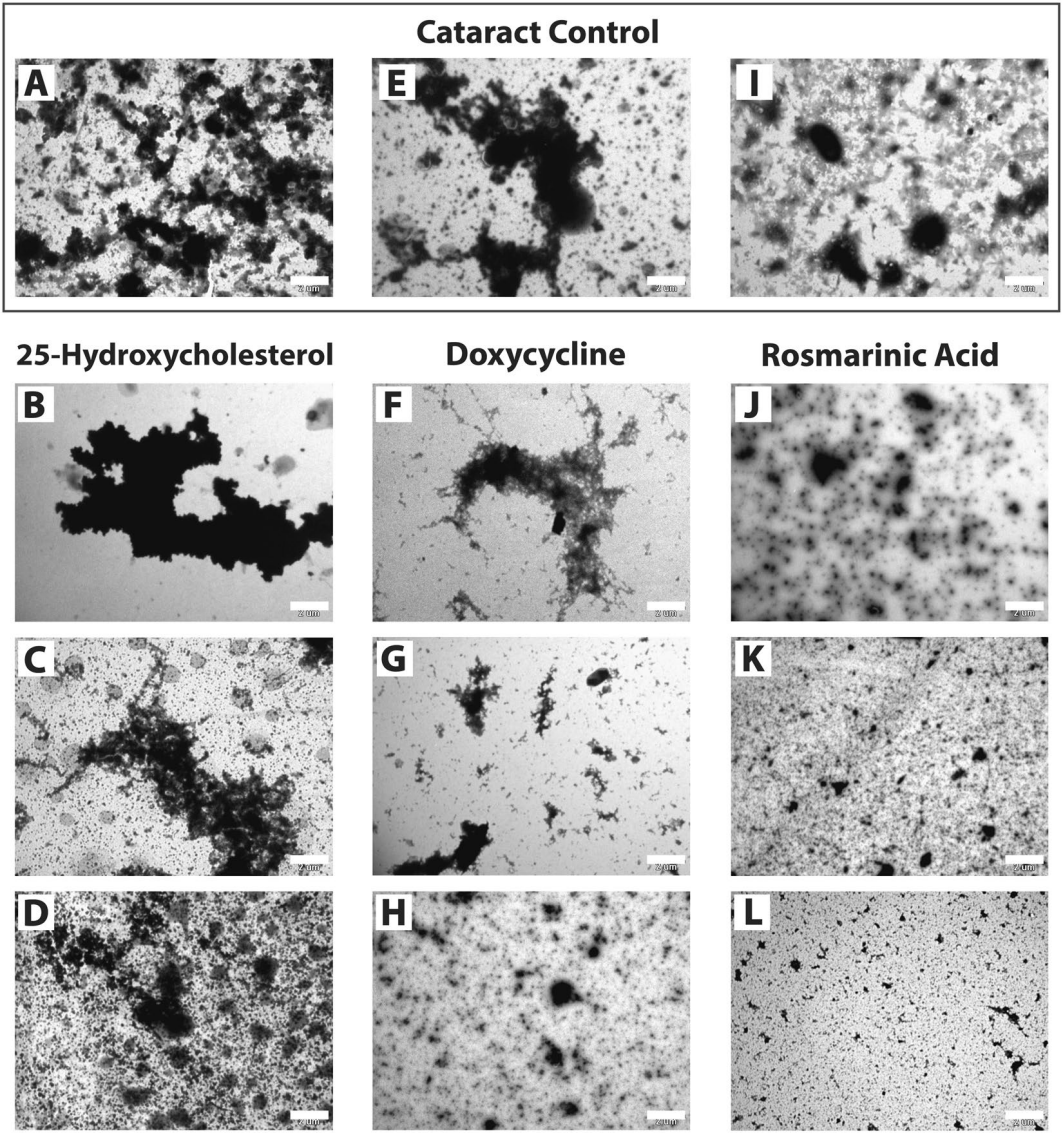
Transmission electron microscopy of cataract solutions treated with 25-hydroxycholesterol, doxycycline, and rosmarinic acid. TEM micrographs of cataract particles in BSS in the absence (**A**,**E**,**I**) or presence of increasing concentrations (50, 500 μM, and 1 mM) of 25-hydroxycholesterol (**B**–**D**) doxycycline (**F**–**H**), or rosmarinic acid (**J**–**L**). Reaction mixtures were incubated for 2 days as described, negatively stained and imaged. Scale bars, 2 µm. Representative electron micrographs from three different experiments conducted with each compound are shown.

**Figure 5:**
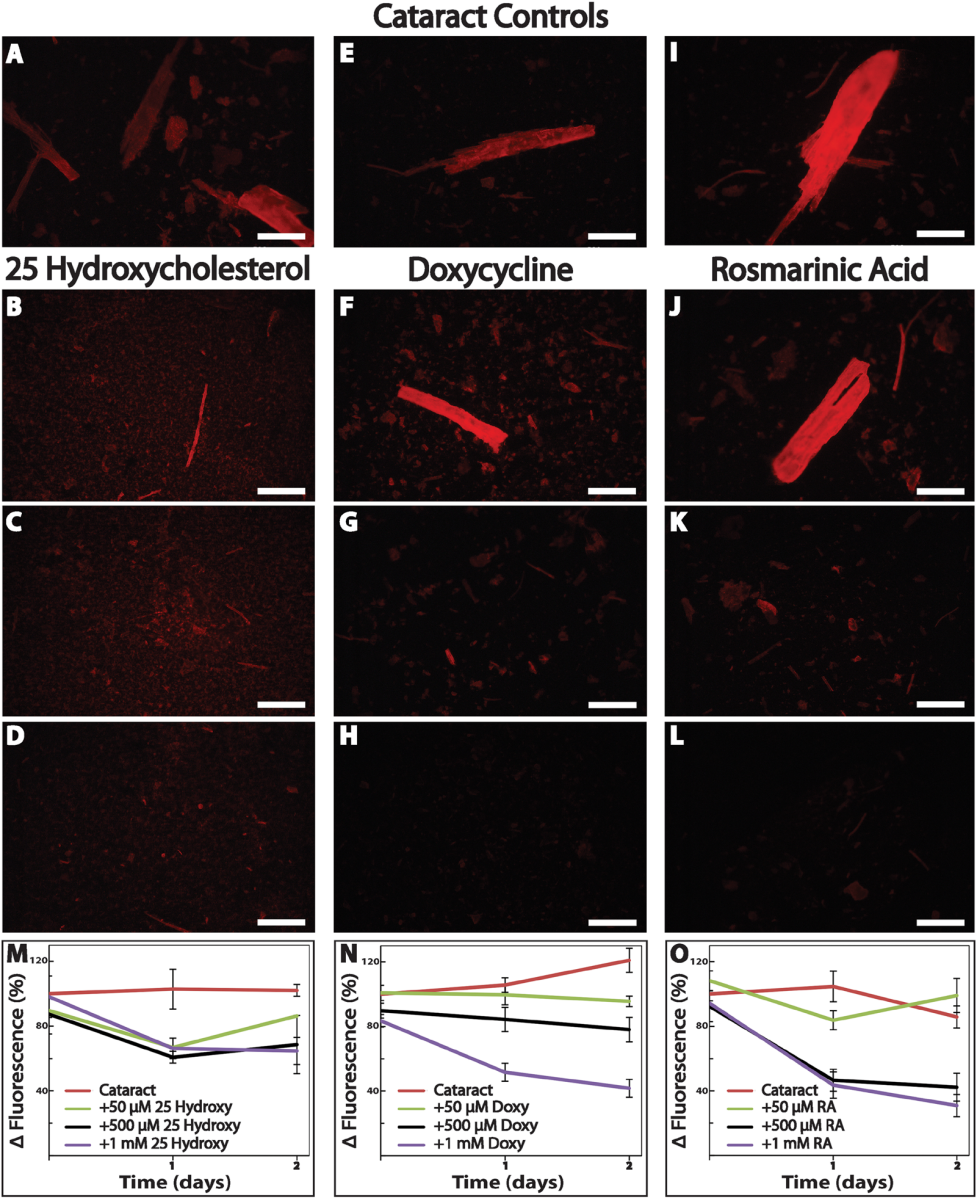
Doxycycline and rosmarinic acid disassemble the amyloidogenic portion of human cataract *ex vivo* in a better manner than 25-hydroxycholesterol. Fluorescence microscopy images of cataract particles in 10% DMSO (**A**–**D**) or pure BSS (**E**–**L**), in the absence (**A**,**E**,**I**) or presence of increasing concentrations (50, 500 μM, and 1 mM) of 25-hydroxycholesterol (**B**–**D**), doxycycline (**F**–**H**), or rosmarinic acid (**J**–**L**). Fluorescence microscopy images were taken one day after the addition of the Congo Red reagent (Excitation and emission wavelength of 540/25 nm and 605/55 nm, respectively). Scale bars, 500 µm. Representative images of five experiments shown. ThT fluorescence kinetics of dispersed cataract in the absence or presence of increasing concentrations of 25-hydroxycholesterol (**M**), doxycycline (**N**), or rosmarinic acid (**O**) were carried out. ThT fluorescence was measured daily for two days and relative fluorescence values are shown. 25-hydroxycholestreol (**M**, 1 mM; n = 7, *P < 0.001), doxycycline (**N**, 1 mM; n = 10, **P = 0.01), and rosmarinic acid (**O**, 500 µM; n = 10, **P < 0.001, 1 mM; n = 10, ***P < 0.001) reduced cataract turbidity in a dose-dependent and significant manner.

Various crystallin proteins are widely known to form amyloids *in vitro*^9,32–34^. Hence, we sought to characterize whether protein assemblies present in the cataract samples possess an ordered structure, and to explore how the cataract-modulating agents affect the observed morphology. To address these questions, we employed transmission electron microscopy analysis. Amorphous and disorganized aggregates were observed for the non-treated cataract solutions (Fig. 4A,E,I). Scanning electron microscopy with energy dispersive X-ray spectroscopy (SEM/EDX) confirmed that these were indeed protein based structures, exhibiting the presence of carbon atoms (Fig. S3). Similar findings were evident from cataract samples treated with 50 µM–1 mM of 25-hydroxycholesterol (Fig. 4B–D). Interestingly, in the presence of 500 µM–1 mM doxycycline (Fig. 4G,H) or 50 µM–1 mM rosmarinic acid (Fig. 4J–L), the size of the amorphous cataract particles was reduced to smaller debris. Consistently with the results of the turbidity assay (Fig. 2), treatment of human cataract with rosmarinic acid resulted in the most noticeable disintegration effect. Control solutions of 25-hydroxycholesterol, doxycycline, or rosmarinic acid examined under the same conditions did not demonstrate any structural or ordered characteristics. Altogether, these findings correlate with our turbidity (Fig. 2) and light microscopy results (Fig. 3), suggesting that both doxycycline and rosmarinic acid possess a cataract modulating effect. Given that both compounds are known amyloid modulators^21,29^, we hypothesized that the cataract modulation effect can be at least partially attributed to the disassembly of its ordered structures. To examine this hypothesis we performed amyloid-specific staining with Congo red and Thioflavin T (ThT) (Fig. 5). Specifically, we sought to determine whether the hallmark characteristics of amyloids are observed in the cataractous material, and how these are altered upon treatment with 25-hydroxycholesterol, doxycycline, or rosmarinic acid.

Indeed, Congo red staining of sonicated human cataract samples resulted in the bright red signal typical for amyloid-specific structures (Fig. 5A,E,I). The intensity of the dye decreased upon two day incubation of the cataract samples with either 25-hydroxycholesterol (1 mM, Fig. 5D), doxycycline (500 µM or 1 mM, Fig. 5G,H), or rosmarinic acid (500 µM or 1 mM, Fig. 5K,L), suggesting that all three compounds are associated with a reduction in the amyloid content present in the phacoemulsified lens particles. In contrast, no fluorescent signal was observed from control solutions containing proteins from sonicated clear bovine crystalline lenses (Fig. S4). Similarly, none of the control solutions containing 25-hydroxycholesterol, doxycycline, or rosmarinic acid in equivalent concentrations examined under the same conditions demonstrated any fluorescent signal.

To validate these results, we performed kinetic ThT fluorescence assays (Fig. 5M–O). We found that non-treated cataract samples that served as controls presented spectra typical for amyloids, with an emission signal at 480 nm when excited at 430 nm (Fig. S5). Moreover, the ThT-fluorescence signal intensity was maintained over two days, indicating the high stability of the fibrillar amyloid content. Among the tested modulating compounds, 25-hydroxycholesterol had the lowest effect on the ThT fluorescence signal intensity of the treated cataract solutions versus the control non-treated cataract sample (Fig. 5M, 1 mM, n = 7, *P < 0.001). A more marked reduction of signal intensity was observed for cataract solutions treated with either doxycycline or rosmarinic acid at 1 mM (Fig. 5N, n = 10, **P = 0.01; Fig. 5O, n = 10, ***P < 0.001, respectively), with the latter also being active at a lower concentration (Fig. 5O, 500 µM, n = 10, **P < 0.001). Taken together, and under the assumption that phacoemulsification does not induce aggregation for itself, our results may indicate that amyloid fibrils are present in human cataract, and that treatment with the aggregation modulators leads to reduction of amyloid-specific signals, consistent with the decrease in the amyloid content.

To study the structural effects of the most potent cataract modulating compound, we performed circular dichroism analysis. This technique allows the overall assessment of the level of secondary structures within the studied sample. When rosmarinic acid was added to *ex-vivo* human cataract samples, a clear dose-dependent reduction in secondary structure content was observed (Fig. S6A). The results clearly indicate a reduction in β-sheet content as reflected in the reduction of the single negative peak at around 218 nm (as compared to the typical double minima at 208 nm and 222 nm for α-helical structures)^35^. Similarly, rosmarinic acid demonstrated a time-dependent effect on the release of soluble proteins from cataract particles, with a 40% and 115% increase in total protein concentration measured one and two days following treatment, respectively (Fig. S6B). Altogether, these observations suggest that treatment with rosmarinic acid is associated with reduction of the beta-sheet content in phacoemulsified human cataract fragments, and with an increase in the soluble protein fraction in the treated solutions. Assuming that the phacoemulsification did not intrinsically stimulate aggregation of the cataractous lens proteins, our findings suggest that treatment of cataract particles with amyloid modulators may induce disaggregation of the amyloid content present in the phacoemulsified cataract, and release of soluble proteins from the insoluble aggregates.

#### *In vivo* animal studies

**Figure 6:**
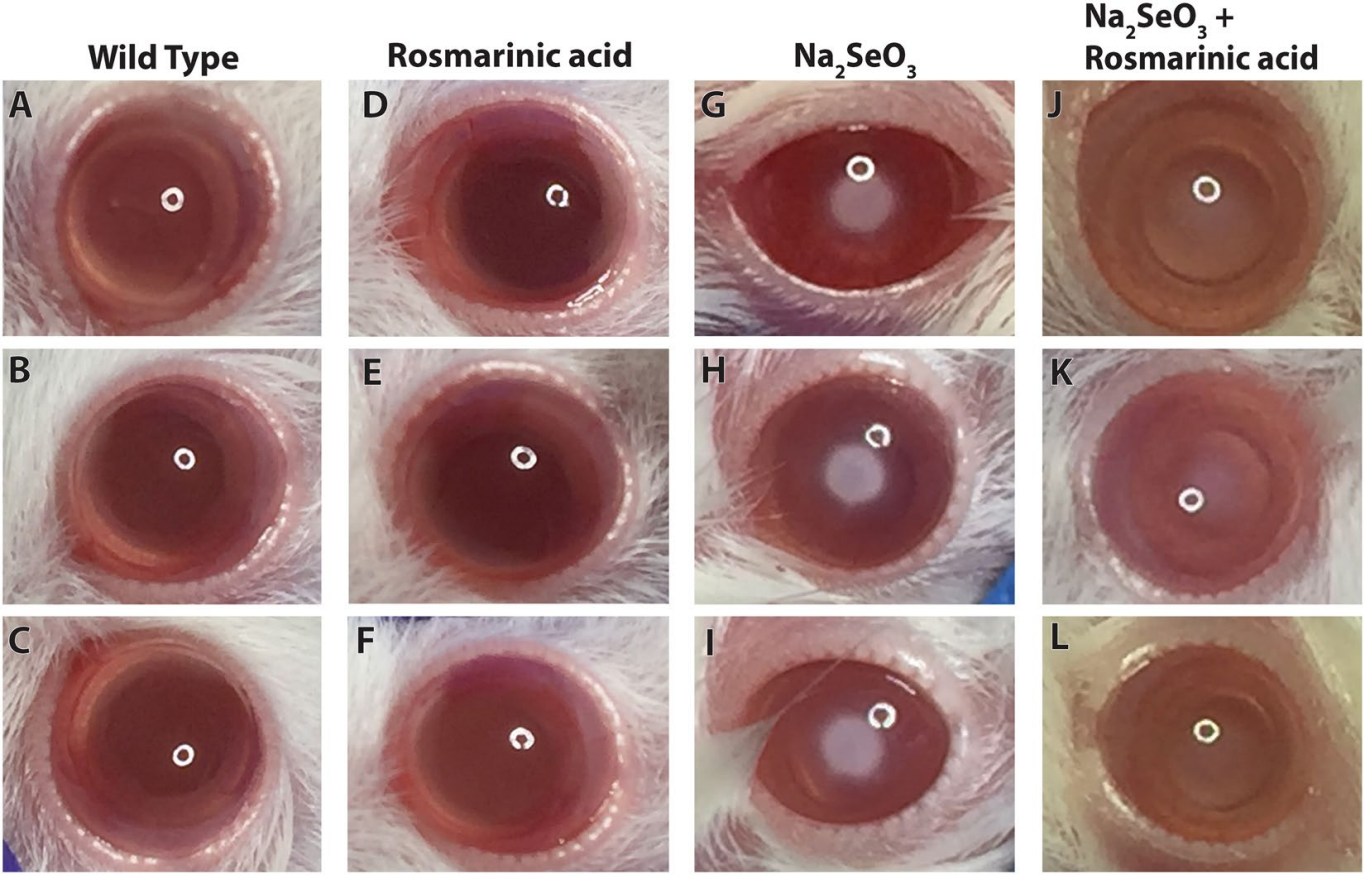
Rosmarinic acid increases lens clarity and delays selenite-induced cataract formation *in vivo*. (**A**–**C**) photographs of wild type rats demonstrating fully transparent lenses and (**D**–**F**) photographs of wild type rats with clear lenses treated with rosmarinic acid. (**G**–**I**) Cataractous lenses from rats treated with postnatal injection of selenite at the age of 13 days. Four days after the injection, a dense opacity in the center of the lens was seen in each of the model eyes. (**J**–**L**) Photographs of model rats treated with Rosmarinic acid. Approximately 4 hours before administration of selenite, the study rats were treated with a subcutaneous injection of rosmarinic acid (40 mM, 500 µl), and were thereafter treated daily with a repeat rosmarinic acid administration. Control rats were treated with a similar protocol but received a subcutaneous injection of a similar volume of PBS. The photographs were acquired six days after the injection. *Rosmarinic acid* treatment delayed lenticular opacification and decreased the severity of selenite-induced cataract in all treated rats.

To test the *in vivo* efficacy of the observed aggregation inhibition on the reduction of lenticular opacity, we employed a pre-clinical rat model of cataract. Newborn rat pups treated with a subcutaneous injection of selenium are known to rapidly develop bilateral lens opacities similar to age-related human cataract^36,37^. Rosmarinic acid, administered subcutaneously 4 hours prior to selenium injection, and daily thereafter, remarkably delayed the appearance of lenticular opacification and reduced cataract severity in treated rats (Fig. 6). The notable treatment effect was evident upon visual inspection as well as by photo grading of cataract stages^38^. Control rat eyes with selenite-induced cataract demonstrated an average lens opacification score of 5, higher by at least 2-grades compared to the study eyes treated with rosmarinic acid (Fig. 6).

## Discussion

The effect of various systemic^10–13^ and topical^14–17^ pharmacological agents on cataract-associated protein aggregation has been investigated in recent years. Several *in vitro* studies have used different purified crystallin proteins and have shown that their aggregation is inhibited by selected small molecules^13,39–41^. Other studies utilized whole or homogenized lenses derived from various animal models, in an attempt to better mimic the pathological conditions in the living human eye^11,42,43^. Makley *et al*. were the first to report that 25-hydroxycholesterol increased the fraction of soluble protein in human lens material as assessed by the bicinchoninic acid assay^16^. Their original results were published whilst we were developing the human *ex vivo* method reported here.

We identified rosmarinic acid as the most potent compound among the small molecules screened for their cataract modulating effect. It was associated with full restoration of the transparency of the sonicated cataractous solution, and demonstrated the highest level of efficacy in all *in vitro* assays performed. Interestingly, Velpandian *et al*. recently reported that a polyherbal eye drop containing rosmarinic acid and other compounds inhibited the progression of cataract in selenite and galactose induced animal cataract models^44^. Rosmarinic acid is a naturally occurring polyphenol present in several herbs, with known antioxidative, anti-cholinergic, anti-inflammatory, and anti-amyloidogenic activities^45–48^. Specifically, it has been shown to bind to amyloid-beta and alpha-synuclein aggregates associated with neurodegenerative diseases^48,49^, and interfere directly with early formed oligomers both *in vitro* and *in vivo*^50,51^. We suggest that rosmarinic acid may lead to restoration of the transparency of the human lens in a similar fashion by binding to cataract-associated proteins present in the phacoemulsified preparations, and reversing their aggregation.

This study has several limitations. The main limitation of this study is that we cannot completely rule out a potentially aggregation-driving impact of phacoemulsification on the cataractous lens proteins in the examined samples. Although the cataractous material samples procured in this study were only briefly exposed to sonication, as the overall effective phacoemulsification time in the hands of experienced surgeons typically ranges between 15–30 seconds or less, the potential impact of phacoemulsification towards induction of protein aggregation cannot be excluded. However, each lens segment that is fragmented and removed from the eye is individually exposed for only several seconds, at most. As such, we believe that the sonication to which the cataract material was exposed during surgery would have played a minor role, if any, in contributing to the formation of amyloid fibrils, seeds or aggregates. Indeed, sonication is a well-established method for disintegration of cells and purification of soluble proteins often employed as an essential step in solubilizing procured cataractous fragments^52–58^. Nonetheless, the possibility that the aggregates present in the soluble preparations in our assay at least partially represent aggregation provoked by the sonication, cannot be ignored. Another limitation is that the selenium induced cataract rat model used here to assess the efficacy of the compounds may not correctly mimic the age-related human cataract in terms of its amyloid content. As such, the solution clarity achieved by rosmarinic acid may have resulted from an effect on additional aggregate structures that do not contain amyloid.

In summary, we have presented a novel *ex vivo* assay that enables to directly test the impact of the examined compounds on the transparency of actual human crystalline lens material. First, we confirmed that 25-hydroxycholesterol indeed reduces cataractous protein load *ex vivo*, supporting the validity of our assay. Next, a screen of various amyloid modulators, to assess their activity in restoration of cataractous lens particles transparency, led to the identification of rosmarinic acid and doxycycline as potent cataract-modifying agents. We report rosmarinic acid as the first compound to restore full transparency in human cataractous lens material and demonstrate its high efficacy in remodeling lenticular protein aggregates. Additional *in vitro* assays, commonly employed in the field of amyloid research, were utilized to provide biophysical characterization of treated *ex vivo* cataractous material, and the efficacy of the lead compound identified by the assay was demonstrated *in vivo* in a rat cataract model. Thus, our novel *ex vivo* assay provides a valuable yet relatively simple model system enabling to directly test the impact of aggregation modulators on human cataract in a comparative manner. This comprehensive platform may be useful for large scale screening for suitable treatment of human cataract. Future studies characterizing the effect of these compounds on purified lens crystallin amyloids *in vitro* may be of interest.

Taken together, our results support the concept that aggregation inhibition may provide a pharmacological treatment strategy for cataract, and highlight rosmarinic acid as a promising potential for palliative treatment of cataract, the leading cause of blindness worldwide. Data obtained from carefully designed and conducted future clinical studies may provide a strong foundation for design of the proper treatment regimen, dosage and duration. We hope that in the near future this promising pharmacological treatment will provide a safe, inexpensive and easily-accessible therapy for cataract.

## Online Methods

### Study Participants

This study was approved by the Institutional Review Board of the Rambam Health Care Campus and all experiments were performed in accordance with relevant guidelines and regulations. Informed consent was obtained from all subjects before participation in the study. Included in this study were patients that were candidates for routine cataract surgery with a nuclear cataract of grade 2 or higher in terms of nuclear opalescence or nuclear color based on the Lens Opacities Classification System III^59^. In all cases, clear corneal incision phacoemulsification cataract surgery was performed at the Department of Ophthalmology, Rambam Health Care Campus, Haifa, Israel.

### Cataract samples collection and handling

Human cataractous nuclear fragments were retrieved from the phacoemulsification hand piece tubing filter following surgery and immediately stored at −20 °C. Prior to each experiment, each nuclear fragment was homogenized in 5 mL of BSS (BVI, Italy) and sonicated for 5 minutes at 40 KHz (Ultrasonic Cleaner 1200H, MRC Labs, Israel). Sodium azide (0.005%) was added as a biocide. Samples were kept either on ice or at −20 °C until used. The β-sheet conformation of the aggregated lenticular material was validated using circular dichroism (Fig. S5).

### Bovine sample collection and handling

Bovine clear lens were retrieved by performing whole lens extraction and were immediately stored at −20 °C. Prior to each experiment, each fragment was homogenized in 5 mL of BSS (BVI, Italy) and sonicated for 5 minutes at 40 KHz (Ultrasonic Cleaner 1200 H, MRC Labs, Israel). Sodium azide (0.005%) was added as a biocide. Samples were kept either on ice or at −20 °C until used.

### Chemicals and Reagents

All chemicals and reagents were of analytical grade (purchased from Sigma-Aldrich, USA).

### *Ex vivo* lens crystalline turbidity analysis

The disaggregation of human cataract samples was monitored by absorbance reading at λ = 340 nm. Total protein concentration was quantified in each cataract sample using the Bradford assay^42,60^. The mean ± SD of the optical density of each sample measured at baseline was 0.50 ± 0.08. All samples were then optimized to a starting point of an optical density ranging between 0.30 to 0.80 a.u. (λ = 340 nm). Samples were then incubated in transparent 96-well plates in triplicates, each consisting of 100 µL *ex vivo* cataract dispersed in BSS containing either 10% vehicle (PBS 1x, pH 7.4 or 10–100% DMSO in PBS, pH 7.4) or increasing concentrations of the tested compounds dissolved in the corresponding vehicle. Plates were sealed with clear sealing tape (Thermo, Denmark) and transferred immediately to an EL808 plate reader (BioTek, USA) at 37 °C. Disaggregation was monitored daily up to two days. In between measurements plates were kept sealed at 37 °C with constant shaking. Compounds that absorb in the visible region of the electromagnetic spectrum were tested up to the highest possible concentration within the spectroscopic limitations of the assay (<a.u. 2.0), while the concentrations of the tested transparent materials varied according to the obtained results. A similar set of samples including only the tested compounds in equivalent concentrations was measured in parallel, for background turbidity subtraction. Turbidity values of the entire experimental set were also recorded at λ = 600 nm, where none of the examined compounds possesses residual absorption properties. In agreement with the results obtained at λ = 340 nm, rosmarinic acid, doxycycline and 25-hydroxycholesterol demonstrated the same patterns of activity of reduction in turbidity (P = 0.02, P = 0.03 and P = 0.001 respectively).

### Capillary assay

Aliquots of cataractous solution in the absence or presence of 50 µM, 500 µM or 1 mM of 25-hydroxycholesterol (in 10% DMSO in PBS 1x, pH 7.4), doxycycline, or rosmarinic acid (in PBS 1x, pH 7.4) after two days of co-incubation were inserted into rectangular glass capillaries (CM Scientific, UK), sealed and observed using bright field microscopy.

### Transmission electron microscopy

Aliquots of 10 µL of cataractous solution in the absence or presence of 50 µM, 500 µM or 1 mM of 25-hydroxycholesterol, doxycycline, or rosmarinic acid (in MOPS 5 mM, pH 7.2) after two days of co-incubation were placed on 400-mesh copper grids. After two minutes, excess fluids were removed and samples were negatively stained with 2% uranyl acetate in water for 30 seconds, blotted with filter paper, and dried overnight. Samples were then analyzed by a JEOL 1200EX electron microscope operating at 80 kV.

### Congo red fluorescence assay

Aliquots of 10 µL of cataractous solution in the absence or presence of 50 µM, 500 µM or 1 mM of 25-hydroxycholesterol (in 10% DMSO in PBS 1x, pH 7.4), doxycycline, or rosmarinic acid (in PBS 1x, pH 7.4) after two days of co-incubation were allowed to dry on a glass microscope slide at room temperature. Samples were then stained by the addition of 10 µl of Congo red staining solution (1:1 v/v ratio in 5 mM phosphate, 150 mM NaCl, pH 7.5, and saturated amount of Congo red), and allowed to dry at room temperature. The stained samples were visualized using a fluorescence microscope (Nikon ECLIPSE E600, USA) at excitation and emission wavelengths of 540/25 nm and 605/55 nm, respectively. We verified that the excitation wavelengths of the dye did not coincide with the wavelength of the peak absorption of the examined compounds.

### Kinetic Thioflavin T (ThT) disassembly assay

To allow the *ex vivo* cataractous solution to homogenously bind to the ThT dye, the dispersed samples were first divided in black 96-well flat-bottomed plates containing ThT solution (20 μM) in PBS (1x, pH 7.4) creating quadricates of 190 μL total volume. Plates were then sealed with clear sealing tape (Thermo, Denmark) and incubated overnight at 37 °C with constant shaking. On the next day, 10 μL of freshly prepared stock solutions of 20 mM, 10 mM, and 1 mM 25-hydroxycholesterol (in DMSO), doxycycline or rosmarinic acid (in PBS 1x, pH 7.4), were added to the cataractous solutions, yielding in total volume of 200 μL. The plates were sealed and incubated at 37 °C with constant shaking for two days. The fluorescence of amyloid-bound ThT was measured daily using CLARIO star plate reader (BMG LABTECH, Germany, excitation and emission wavelength of 430 nm and 492 nm, respectively). A similar set of samples including the same amounts of the tested compounds was prepared in BSS and measured in parallel for background fluorescence subtraction. We verified that the excitation wavelengths of the dye did not coincide with the wavelength of the peak absorption of the examined compounds. Relative fluorescence was calculated by subtracting the signals of the vehicle and small molecules controls from those of the samples. The results are displayed as percentages of the fluorescence of the untreated cataractous solutions for each time point. In between measurements, plates were kept sealed at 37 °C with constant shaking.

### Statistical analysis

Data were analyzed using the Minitab Software, version 17 (Minitab Inc, State College, PA). Turbidity and ThT disassembly over time were analyzed using repeated-measures analysis of variance followed by Bonferroni correction for multiple paired comparisons. A *P*-value of less than 0.1 was considered statistically significant.

### Animal studies

Injection of sodium selenite (Na_2_SeO_3_) in rats 10–18 days old is known to rapidly induce lens opacification similar to the nuclear and cortical age-related human cataract phenotypes^36,37^. Newborn Wistar male and female rat pups were treated at postnatal age of 13 days with a single subcutaneous injection of sodium selenite (30 µmol/kg body weight). Approximately 4 hours before administration of selenite, the study rats were treated with a subcutaneous injection of rosmarinic acid (40 mM, 500 µl), and were thereafter treated daily with a repeat rosmarinic acid administration. Control rats were treated with a similar protocol, but received a subcutaneous injection of a similar volume of PBS. All rats were monitored daily for the development of cataract. Assessment of the lenticular clarity *in vivo* was performed by visual inspection under a binocular microscope following brief anesthesia by inhalation of Isoflurane 3%, and administration of mydriatic eye drops. The degree of the cataract severity was assessed according to previously published grading systems^38^ by two trained ophthalmologists who independently assessed photographs of the treated and control eyes. The study was approved by the ethics committee of the Ruth and Bruce Rappaport Faculty Of Medicine, Technion Israel Institute of technology, Haifa, Israel and all experiments were performed in accordance with relevant guidelines and regulations.

### Data availability

The authors declare that the data supporting the findings of this study are available within the paper and its supplementary information files.

## Electronic supplementary material

Supplementary Materials

## References

[1] Pascolini D, Mariotti SP (2012). Global estimates of visual impairment: 2010. The British journal of ophthalmology.

[2] Frick KD, Foster A (2003). The magnitude and cost of global blindness: an increasing problem that can be alleviated. American journal of ophthalmology.

[3] Lamoureux EL, Fenwick E, Pesudovs K, Tan D (2011). The impact of cataract surgery on quality of life. Current opinion in ophthalmology.

[4] Organization, W. H. Vision 2020 The Right to Sight (2006).

[5] Lundstrom M, Barry P, Henry Y, Rosen P, Stenevi U (2012). Evidence-based guidelines for cataract surgery: guidelines based on data in the European Registry of Quality Outcomes for Cataract and Refractive Surgery database. Journal of cataract and refractive surgery.

[6] Shoss BL, Tsai LM (2013). Postoperative care in cataract surgery. Current opinion in ophthalmology.

[7] Takemoto L, Sorensen CM (2008). Protein-protein interactions and lens transparency. Experimental eye research.

[8] Sandilands A (2002). Altered aggregation properties of mutant gamma-crystallins cause inherited cataract. The EMBO journal.

[9] Meehan S (2004). Amyloid fibril formation by lens crystallin proteins and its implications for cataract formation. The Journal of biological chemistry.

[10] Sreelakshmi V, Sasikala V, Abraham A (2015). Luteolin Supplementation Prevents Selenite-Induced Cataractogenesis in Sprague Dawley Rat Pups. Chemistry & biodiversity.

[11] Liao JH (2016). Anticataractogenesis Mechanisms of Curcumin and a Comparison of Its Degradation Products: An *in Vitro* Study. Journal of agricultural and food chemistry.

[12] Sasikala V, Rooban BN, Sahasranamam V, Abraham A (2013). Rutin ameliorates free radical mediated cataract by enhancing the chaperone activity of alpha-crystallin. Graefe’s archive for clinical and experimental ophthalmology = Albrecht von Graefes Archiv fur klinische und experimentelle Ophthalmologie.

[13] Nahomi RB (2013). Chaperone peptides of alpha-crystallin inhibit epithelial cell apoptosis, protein insolubilization, and opacification in experimental cataracts. The Journal of biological chemistry.

[14] Varma SD, Kovtun S, Hegde K (2010). Effectiveness of topical caffeine in cataract prevention: studies with galactose cataract. Molecular vision.

[15] Zhao L (2015). Lanosterol reverses protein aggregation in cataracts. Nature.

[16] Makley LN (2015). Pharmacological chaperone for alpha-crystallin partially restores transparency in cataract models. Science.

[17] Kador PF, Betts D, Wyman M, Blessing K, Randazzo J (2006). Effects of topical administration of an aldose reductase inhibitor on cataract formation in dogs fed a diet high in galactose. American journal of veterinary research.

[18] Shanmugam PM (2015). Effect of lanosterol on human cataract nucleus. Indian journal of ophthalmology.

[19] Young LM (2015). Screening and classifying small-molecule inhibitors of amyloid formation using ion mobility spectrometry-mass spectrometry. Nature chemistry.

[20] Familian A, Boshuizen RS, Eikelenboom P, Veerhuis R (2006). Inhibitory effect of minocycline on amyloid beta fibril formation and human microglial activation. Glia.

[21] Forloni G, Colombo L, Girola L, Tagliavini F, Salmona M (2001). Anti-amyloidogenic activity of tetracyclines: studies *in vitro*. FEBS letters.

[22] Cohen T, Frydman-Marom A, Rechter M, Gazit E (2006). Inhibition of amyloid fibril formation and cytotoxicity by hydroxyindole derivatives. Biochemistry.

[23] Bieschke J (2010). EGCG remodels mature alpha-synuclein and amyloid-beta fibrils and reduces cellular toxicity. Proceedings of the National Academy of Sciences of the United States of America.

[24] Sheynis T (2013). Aggregation modulators interfere with membrane interactions of beta2-microglobulin fibrils. Biophysical journal.

[25] Bastianetto S, Yao ZX, Papadopoulos V, Quirion R (2006). Neuroprotective effects of green and black teas and their catechin gallate esters against beta-amyloid-induced toxicity. The European journal of neuroscience.

[26] Ono K, Hasegawa K, Naiki H, Yamada M (2004). Anti-amyloidogenic activity of tannic acid and its activity to destabilize Alzheimer’s beta-amyloid fibrils *in vitro*. Biochimica et biophysica acta.

[27] Scherzer-Attali R (2010). Complete phenotypic recovery of an Alzheimer’s disease model by a quinone-tryptophan hybrid aggregation inhibitor. PloS one.

[28] Scherzer-Attali R, Shaltiel-Karyo R, Adalist YH, Segal D, Gazit E (2012). Generic inhibition of amyloidogenic proteins by two naphthoquinone-tryptophan hybrid molecules. Proteins.

[29] Iuvone T, De Filippis D, Esposito G, D’Amico A, Izzo AA (2006). The spice sage and its active ingredient rosmarinic acid protect PC12 cells from amyloid-beta peptide-induced neurotoxicity. The Journal of pharmacology and experimental therapeutics.

[30] Ono K, Hasegawa K, Naiki H, Yamada M (2004). Curcumin has potent anti-amyloidogenic effects for Alzheimer’s beta-amyloid fibrils *in vitro*. Journal of neuroscience research.

[31] Ono K, Yamada M (2006). Antioxidant compounds have potent anti-fibrillogenic and fibril-destabilizing effects for alpha-synuclein fibrils *in vitro*. Journal of neurochemistry.

[32] Moran SD (2012). Two-dimensional IR spectroscopy and segmental 13C labeling reveals the domain structure of human gammaD-crystallin amyloid fibrils. Proceedings of the National Academy of Sciences of the United States of America.

[33] Papanikolopoulou K (2008). Formation of amyloid fibrils *in vitro* by human gammaD-crystallin and its isolated domains. Molecular vision.

[34] Wang Y (2010). Formation of amyloid fibrils *in vitro* from partially unfolded intermediates of human gammaC-crystallin. Investigative ophthalmology & visual science.

[35] Greenfield NJ (2006). Using circular dichroism spectra to estimate protein secondary structure. Nature protocols.

[36] Shearer TR, David LL, Anderson RS, Azuma M (1992). Review of selenite cataract. Current eye research.

[37] Shearer TR, David LL, Anderson RS (1987). Selenite cataract: a review. Current eye research.

[38] Clark JI, Livesey JC, Steele JE (1996). Delay or inhibition of rat lens opacification using pantethine and WR-77913. Experimental eye research.

[39] Liu Y (2014). Hemin as a generic and potent protein misfolding inhibitor. Biochem Biophys Res Commun.

[40] Liao JH (2014). Carnosine ameliorates lens protein turbidity formations by inhibiting calpain proteolysis and ultraviolet C-induced degradation. Journal of agricultural and food chemistry.

[41] Schafheimer N, King J (2013). Tryptophan cluster protects human gammaD-crystallin from ultraviolet radiation-induced photoaggregation *in vitro*. Photochemistry and photobiology.

[42] Ferlemi AV, Makri OE, Mermigki PG, Lamari FN, Georgakopoulos CD (2016). Quercetin glycosides and chlorogenic acid in highbush blueberry leaf decoction prevent cataractogenesis *in vivo* and *in vitro*: Investigation of the effect on calpains, antioxidant and metal chelating properties. Experimental eye research.

[43] Nagai N, Ito Y (2014). Excessive hydrogen peroxide enhances the attachment of amyloid beta1-42 in the lens epithelium of UPL rats, a hereditary model for cataracts. Toxicology.

[44] Velpandian T, Gupta P, Ravi AK, Sharma HP, Biswas NR (2013). Evaluation of pharmacological activities and assessment of intraocular penetration of an ayurvedic polyherbal eye drop (Itone) in experimental models. BMC complementary and alternative medicine.

[45] Petersen M, Simmonds MS (2003). Rosmarinic acid. Phytochemistry.

[46] Osakabe N, Yasuda A, Natsume M, Yoshikawa T (2004). Rosmarinic acid inhibits epidermal inflammatory responses: anticarcinogenic effect of Perilla frutescens extract in the murine two-stage skin model. Carcinogenesis.

[47] Perry EK, Pickering AT, Wang WW, Houghton PJ, Perry NS (1999). Medicinal plants and Alzheimer’s disease: from ethnobotany to phytotherapy. The Journal of pharmacy and pharmacology.

[48] Camilleri A (2013). Mitochondrial membrane permeabilisation by amyloid aggregates and protection by polyphenols. Biochimica et biophysica acta.

[49] Yamada M, Ono K, Hamaguchi T, Noguchi-Shinohara M (2015). Natural Phenolic Compounds as Therapeutic and Preventive Agents for Cerebral Amyloidosis. Advances in experimental medicine and biology.

[50] Ono K (2012). Phenolic compounds prevent amyloid beta-protein oligomerization and synaptic dysfunction by site-specific binding. The Journal of biological chemistry.

[51] Airoldi C (2013). Natural compounds against Alzheimer’s disease: molecular recognition of Abeta1-42 peptide by Salvia sclareoides extract and its major component, rosmarinic acid, as investigated by NMR. Chemistry, an Asian journal.

[52] Ortwerth BJ, Olesen PR, Sharma KK (1986). Solubilization of the lens water-insoluble fraction by sonication. Experimental eye research.

[53] Ortwerth BJ, Olesen PR (1989). Studies on the nature of the water-insoluble fraction from aged bovine lenses. Experimental eye research.

[54] Cheng R, Lin B, Ortwerth BJ (2002). Rate of formation of AGEs during ascorbate glycation and during aging in human lens tissue. Biochimica et biophysica acta.

[55] Ortwerth BJ, Olesen PR (1992). Studies on the solubilization of the water-insoluble fraction from human lens and cataract. Experimental eye research.

[56] Linetsky M, Shipova E, Cheng R, Ortwerth BJ (2008). Glycation by ascorbic acid oxidation products leads to the aggregation of lens proteins. Biochimica et biophysica acta.

[57] Sharma KK, Ortwerth BJ (1995). Effect of cross-linking on the chaperone-like function of alpha crystallin. Experimental eye research.

[58] Yanshole LV (2013). Cataract-specific posttranslational modifications and changes in the composition of urea-soluble protein fraction from the rat lens. Molecular vision.

[59] Chylack LT (1993). The Lens Opacities Classification System III. The Longitudinal Study of Cataract Study Group. Archives of ophthalmology.

[60] Bradford MM (1976). A rapid and sensitive method for the quantitation of microgram quantities of protein utilizing the principle of protein-dye binding. Analytical biochemistry.

