# Supplementary material for: Rosmarinic Acid Restores Complete Transparency of Sonicated Human Cataract Ex Vivo and Delays Cataract Formation In Vivo
Source: Sci Rep. 2018 Jun 19;8:9341. doi: 10.1038/s41598-018-27516-9 (PMC6008418; doi:10.1038/s41598-018-27516-9)
Supplement: Supplementary file 1 — Supplementary Materials [file 41598_2018_27516_MOESM1_ESM.docx]

**Rosmarinic Acid Restores Complete Transparency of Sonicated Human Cataract *Ex Vivo* and Delays Cataract Formation *In Vivo***

Marina Chemerovski-Glikman,^¥1^ Michael Mimouni,^¥2^ Yarden Dagan,^1^ Esraa Haj,^1^ Igor Vainer,^2^ Raviv Allon,^2^ Eytan Z. Blumenthal,^2^ Lihi Adler-Abramovich,^3^ Daniel Segal,^1, 4^ Ehud Gazit,^*1, 5^, and Shiri Zayit-Soudry^*2^

^1^Department Molecular Microbiology and Biotechnology, George S. Wise Faculty of Life Sciences, Tel-Aviv University, Tel Aviv, 69978, Israel

^2^Department of Ophthalmology, Rambam Health Care Campus, Technion Israel Institute of Technology, Haifa, Israel

^3^Department of Oral Biology, The Goldschleger School of Dental Medicine, Sackler Faculty of Medicine, Tel Aviv University, Tel Aviv 69978, Israel.

^4^Sagol Interdisciplinary School of Neurosciences, Tel-Aviv University, Tel Aviv, 69978, Israel

^5^Department of Materials Science and Engineering, Iby and Aladar Fleischman Faculty of Engineering, Tel Aviv University, Tel Aviv 6997801, Israel.

^¥^Authors contributed equally, ^*^Corresponding authors

**Supplementary Materials**

**
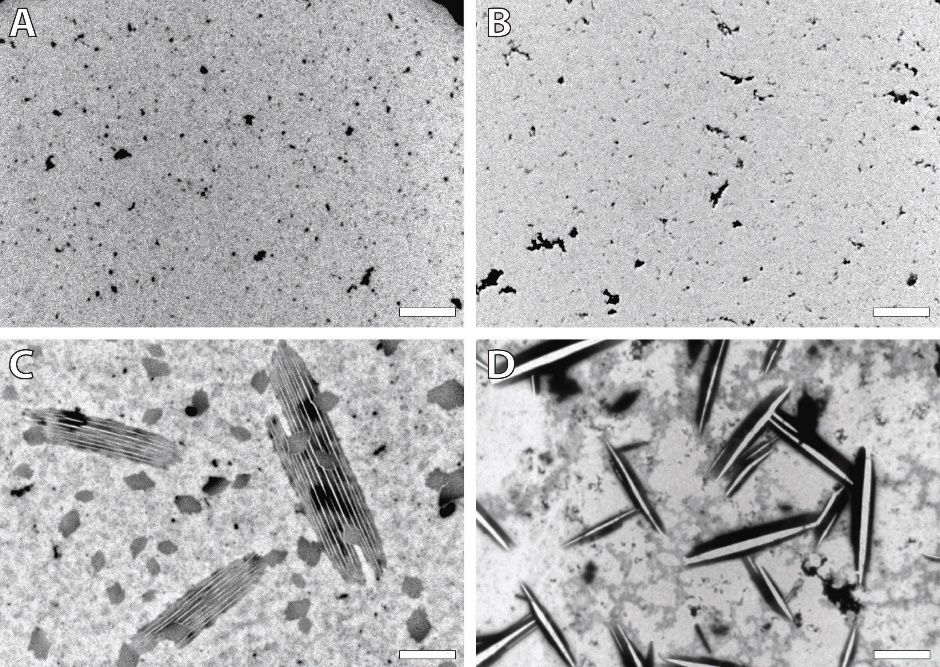
**

**Figure S1**: **Tannic acid possesses an intrinsic tendency to self-assemble into ordered structures**. Transmission electron microscopy (TEM) of tannic acid incubated without cataract solution for 2 days at increasing concentrations of 1 µM (A), 50 µM (B), 200 µM (C) and 500µM (D) . Scale bar: 2 µm. Representative electron micrographs from three different experiments conducted with each compound concentration are shown. At the higher concentrations tested (200 and 500 μM), ordered crystal-like structures were observed.


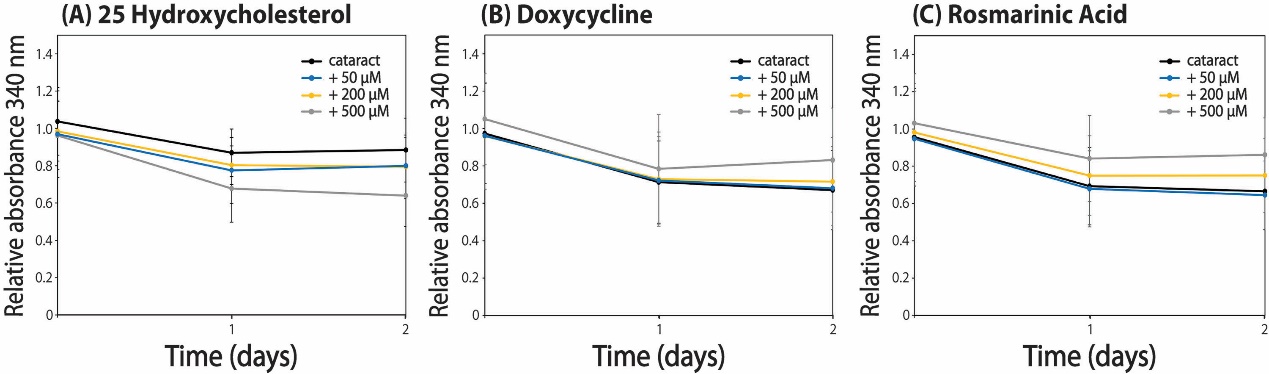


**Figure S2: Clear bovine lens *ex vivo* turbidity experiment.** The effect of the lead compounds on the turbidity of clear bovine lens samples was monitored by absorbance measurement at λ = 340 nm and 37 °C. Solutions containing clear bovine lens particles were incubated with increasing concentrations of (A) 25-hydroxycholesterol, (B) doxycycline, and (C) rosmarinic acid in triplicates, over two days. No significant effect on the turbidity of the treated clear bovine lens samples was observed for either compound. No direct comparison can be made between the relative absorbance of the bovine and the human lens material, since they required different dilutions for optimal absorbance measurements.

**
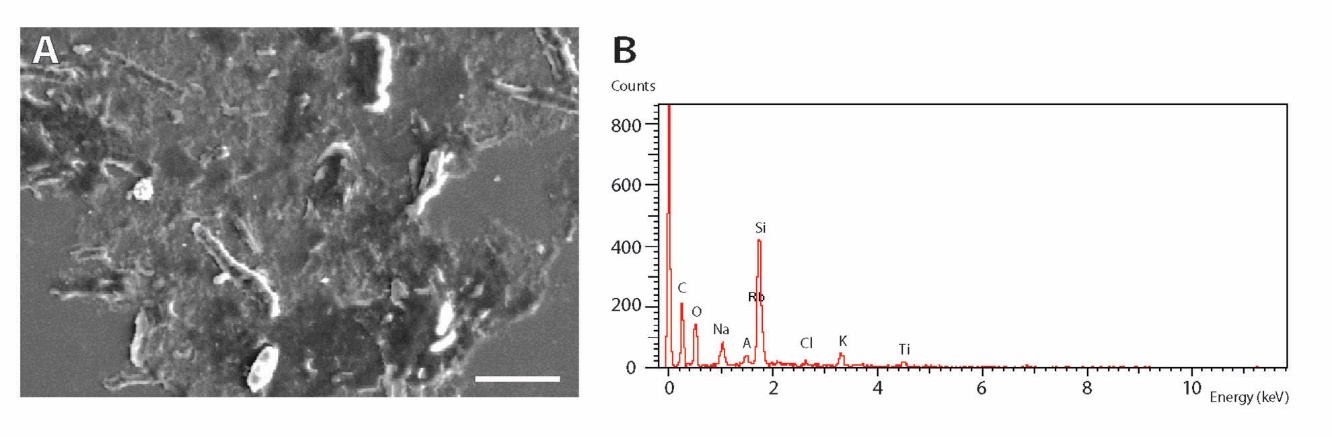
**

**Figure S3:** Scanning electron microscopy with energy dispersive X-ray spectroscopy (SEM/EDX) A. Electron microscopy micrograph of the cataract sample. Scale bar: 20 µm. B. X-ray spectroscopy demonstrating that these structures contain carbon.


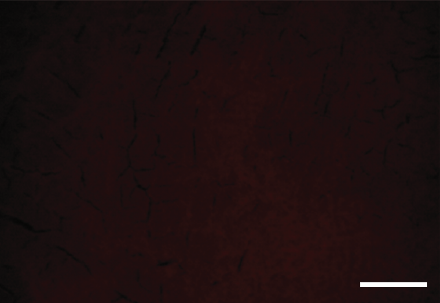


**Figure S4:** Micrograph showing clear bovine lens solution treated with Congo red staining, visualized using a fluorescence microscope. There is no evidence of any fluorescent bright red signal typical for amyloid content. Similar findings were noted in all other clear bovine lens samples. Scale bar: 250 µm.

**
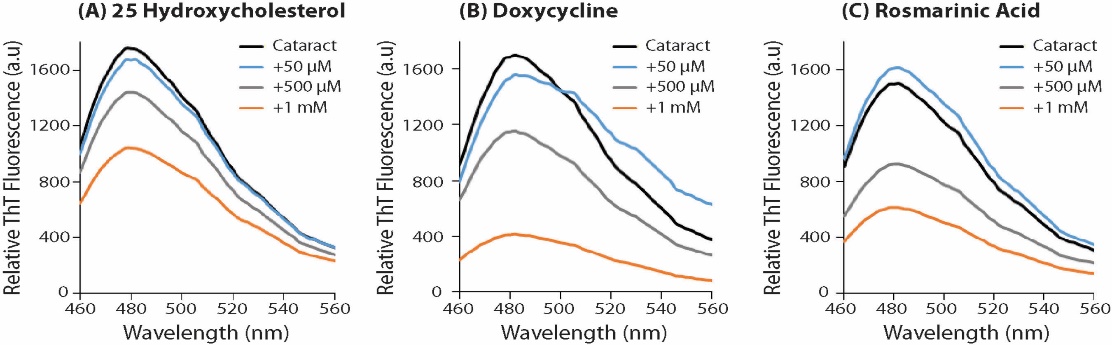
**

**Figure S5**: ThT emission spectra of cataract in the absence and presence of 25-hydroxycholesterol (A, 25-Hydroxy), doxycycline (B, Doxy), and rosmarinic acid (C, RA). Samples were excited at 430 nm.

**
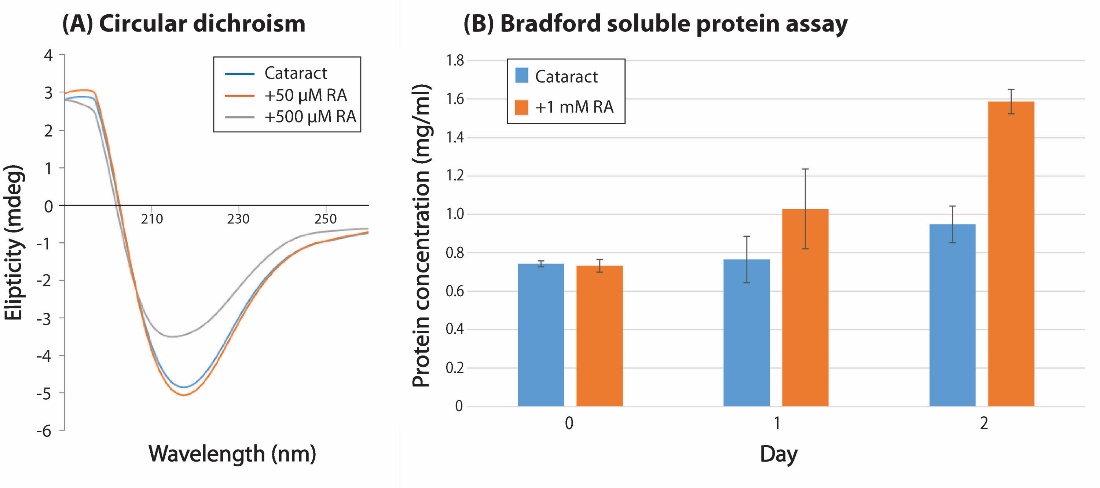
**

**Figure S6**: Secondary structural analysis of cataract samples by circular dichroism demonstrating a typical β-sheet pattern for the cataract samples only. When rosmarinic acid was added a clear dose dependent reduction in secondary structure content was observed (A). Rosmarinic acid demonstrated a time-dependent effect on the release of proteins from cataractous materials with a 40% and 115% increase in total protein concentration one and two days following exposure, respectively (B).
